# Investigating the Impact of Sensor Layout on Radiation Hardness in 25 µm Pitch Hybrid Pixel Detectors for 4th Generation Synchrotron Light Sources

**DOI:** 10.3390/s25113383

**Published:** 2025-05-28

**Authors:** Julian Heymes, Filippo Baruffaldi, Anna Bergamaschi, Martin Brückner, Maria Carulla, Roberto Dinapoli, Simon Ebner, Khalil Ferjaoui, Erik Fröjdh, Viveka Gautam, Dominic Greiffenberg, Shqipe Hasanaj, Viktoria Hinger, Thomas King, Pawel Kozłowski, Shuqi Li, Carlos Lopez-Cuenca, Alice Mazzoleni, Davide Mezza, Konstantinos Moustakas, Aldo Mozzanica, Martin Müller, Jonathan Mulvey, Jan Navrátil, Kirsty A. Paton, Christian Ruder, Bernd Schmitt, Patrick Sieberer, Dhanya Thattil, Xiangyu Xie, Jiaguo Zhang

**Affiliations:** Paul Scherrer Institut, Forschungsstrasse 111, 5232 Villigen, Switzerland; filippo.baruffaldi@dectris.com (F.B.); anna.bergamaschi@psi.ch (A.B.); martin.brueckner@psi.ch (M.B.); maria.carulla@psi.ch (M.C.); roberto.dinapoli@psi.ch (R.D.); simon.ebner@psi.ch (S.E.); khalil.ferjaoui@psi.ch (K.F.); erik.froejdh@psi.ch (E.F.); viveka.gautam@psi.ch (V.G.); dominic.greiffenberg@psi.ch (D.G.); shqipe.hasanaj@psi.ch (S.H.); viktoria.hinger@psi.ch (V.H.); thomas.king@psi.ch (T.K.); pawel.kozlowski@cern.ch (P.K.); shuqi.li@psi.ch (S.L.); carlos.lopez-cuenca@psi.ch (C.L.-C.); alice.mazzoleni@psi.ch (A.M.); davide.mezza@psi.ch (D.M.); konstantinos.moustakas@psi.ch (K.M.); aldo.mozzanica@psi.ch (A.M.); martin.mueller@psi.ch (M.M.); jonathan.mulvey@psi.ch (J.M.); xnavra66@vutbr.cz (J.N.); kirsty.paton@psi.ch (K.A.P.); christian.ruder@psi.ch (C.R.); bernd.schmitt@psi.ch (B.S.); patrick.sieberer@psi.ch (P.S.); dhanya.thattil@psi.ch (D.T.); xiangyu.xie@psi.ch (X.X.); jiaguo.zhang@psi.ch (J.Z.)

**Keywords:** hybrid pixel detectors, pixel sensor design, radiation hardness, irradiation, X-ray detector, sensor layout

## Abstract

With the evolution of synchrotron light sources to fourth generation (diffraction-limited storage rings), the brilliance is increased by several orders of magnitude compared to third generation facilities. For example, the Swiss Light Source (SLS) has been upgraded to SLS 2.0, promising a horizontal emittance reduced by a factor of 40, and a brilliance up to two orders of magnitude (three at higher energies). A key challenge arising from the increased flux is the heightened accumulated dose in silicon sensors, which leads to a significant increase in radiation damage. This translates into an increase of both noise and dark current, as well as a reduction in the dynamic range for long exposure times, thus affecting the performance of the detector, in particular, for charge-integrating detectors. We have designed sensors with a 4 × 4 mm^2^ pixel array featuring 16 design variations of 25 µm pitch pixels with different implant and metal sizes and tested them bump-bonded to MÖNCH 0.3, a charge integrating hybrid pixel detector readout ASIC. Following a first assessment of the functionality and performance of the different pixel designs, the assembly has been irradiated with X-rays. The variation in the tested parameters was characterized at different accumulated doses up to 100 kGy at the sensor entrance window side. The annealing dynamics at room temperature have also been measured. The results show that the default pixel design is currently not optimal and can benefit from layout changes (reduction in the inter-pixel gap area with full metal coverage of the implant). Further studies on the metal coverage over large implants could be conducted. The layout changes are, however, not sufficient for future full-sized sensors, requiring improved radiation hardness and long-term stability, and additional strategies such as focusing on detector cooling and changes in sensor technologies would be required.

## 1. Introduction

Hybrid pixel detectors (HPDs) have been the workhorse detectors at synchrotron and X-ray free electron laser (XFEL) facilities for over two decades. These detectors are composed of sensing elements bump-bonded to one or more readout Application Specific Integrated Circuits (ASICs). The ASICs, available as photon-counting and charge-integrating types, are developed independently from the sensor. Currently, the minimal available pixel pitch for hybrid detectors is 25 µm [1].

The sensor layer can be fabricated out of different semiconductor materials and technologies (e.g., silicon, GaAs:Cr, CdTe, CZT), with silicon being the most common choice due to material quality and availability. In the case of silicon, the sensor is composed of an array of diodes fabricated on a high resistivity substrate with typical thicknesses ranging between 300 µm and 650 µm (up to 1 mm available) for X-ray detection. These sensors are the most efficient for photon energies ranging from approximately 1 keV (limited by the readout noise and the entrance window thickness [2]) to 25 keV (quantum efficiency limited - at this energy, 83.8% of the photons are transmitted through a 320 µm thick sensor). The detection of softer X-rays (<1 keV) can be achieved with HPD using Low Gain Avalanche Diodes (LGADs) sensors [2] with optimized entrance window and gain layer design, while high-Z materials are preferred for hard X-rays with higher energies (>20 keV) [3].

The standard silicon sensors fabricated by sensor foundries are not radiation tolerant by default. Understanding the achievable performance for a given accumulated dose in a sensor and the possible improvements necessary becomes essential for their use at diffraction-limited storage rings (DLSR, fourth generation synchrotron light source [4]). For instance, the Swiss Light Source (SLS) has been upgraded to SLS 2.0 [5], promising a 40-fold reduction in horizontal emittance and, with upgraded optics, some beamlines are expected to have an increase in brilliance of up to two orders of magnitude (up to three at higher energies).

In the following sections, the radiation hardness of different pixel designs is evaluated using the 25 µm pitch MÖNCH 0.3 ASIC prototype. The MÖNCH project and ASIC’s features are presented in Section 2 with an example of an irradiated sensor during a computed tomography experiment (Section 2.1). The physical causes for radiation damage influenced by the layout of the sensor are outlined in Section 2.2. A prototype sensor with 16 different pixel layouts to explore the effects of radiation damage is presented in Section 3 and its irradiation process is presented in Section 4. The noise and dark current performance results are presented and discussed in Section 5. The work is concluded with perspectives towards small pitch radiation-hard HPDs at DLSRs.

## 2. Radiation Damage in Silicon Sensors: A Practical Example

To explore the effects of the sensor layout on radiation tolerance, the MÖNCH 0.3 prototype detector has been selected. The charge-integrating readout ASIC designed in UMC 110 nm technology [6] features a 1 × 1 cm^2^ pixel array of 400 × 400 pixels with 25 µm pitch, and down around 30-35 e^−^ rms equivalent noise charge (ENC) [1]. The MÖNCH project [1,7,8] is currently in its fifth prototyping phase, and is expected to be available as an approximately 2.56 × 1.92 cm^2^ full-scale detector in 2026–2027 [9]. MÖNCH already offers excellent native spatial resolution without interpolation algorithms compatible with applications such as fast phase-contrast computed tomography. The combination of low noise and small pixels means the effects of charge sharing can be exploited with interpolation algorithms to achieve spatial resolution well below the pixel size [10,11]. However, due to X-ray radiation damage to the sensor and ASIC, the detector suffers from increased dark current and noise. The radiation damage in the sensor can be explained by two main accumulation processes (oxide charge and interface traps) [12,13]. An increase in border traps exists as a third process, but is not considered in this paper.

### 2.1. Example of Experiment-Induced Radiation Damage

An *in-situ* phase-contrast computed tomography of mouse lungs was conducted utilizing a MÖNCH 0.3 with a 320 µm thick silicon sensor at the SYRMEP beamline of the Elettra synchrotron in Trieste (Italy) [14,15]. The detector was placed 1.5 m away from the sample and 22 keV monochromatic photons were used (absorption length of 1.26 mm in silicon). The beam covers a wide area, of up to 120 × 4 mm^2^, therefore, uniformly irradiating a large sensor area.

**Figure 1:**
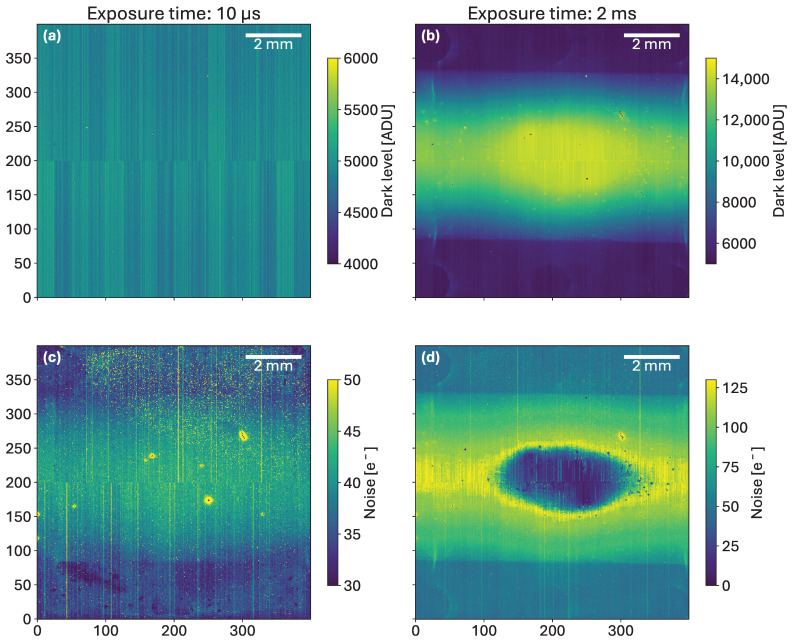
Example of a radiation damaged MÖNCH 0.3 assembly several years after an in-situ phase-contrast computer tomography experiment with 22 keV photons. Top row: Dark frame at an exposure time of (**a**) 10 µs and (**b**) 2 ms. Bottom row: Noise map at an exposure time of (**c**) 10 µs and (**d**) 2 ms. At 2 ms exposure time, the central ovoid irradiated area clearly appears saturated in High Gain/CDS Gain 4.

After multiple days of measurement, radiation damage effects became visible, especially at longer exposure times. Selected frames of the observable damage shown in Figure 1 were acquired several years after the irradiation, indicating extremely slow recovery at room temperature.

The sensor mean dark level (over 1000 frames) in Analog-to-Digital Units (ADU) at a short exposure time (10 µs) is shown in Figure 1a, and at a long exposure time (2 ms) in Figure 1b (such very long exposure is selected to emphasize the effects of the damage in this specific detector assembly). As a comparison, consider that a single 12 keV photon corresponds to approx. 1800 ADU with a conversion gain of 150 ADU/keV. Figure 1c,d shows the noise levels in electrons at the same exposure times. The noise levels at the same exposure times have been measured using a reference unirradiated MÖNCH 0.3: 29.6 ± 1.0 e^−^ at 10 µs exposure time, and 89.6 ± 2.8 e^−^ at 2 ms exposure time.

At a short exposure time, the dark level is uniform, and only a very small variation in noise can be observed within the irradiated area (by limiting the color scale). At a long exposure time, the irradiated area is easily noticeable on both the dark level and the noise image. The beam covered the entire width of the detector and about half the height (centered around the middle).

At the center of the sensor, where the beam was most intense, the noise level seen in the noise image is very low, indicating saturation (as the noise is computed as the standard deviation of the frames). The increase in the dark level and corresponding decrease in noise is mainly due to the increase in dark current due to radiation damage.

### 2.2. Layout Influence on Radiation Damage

**Figure 2:**
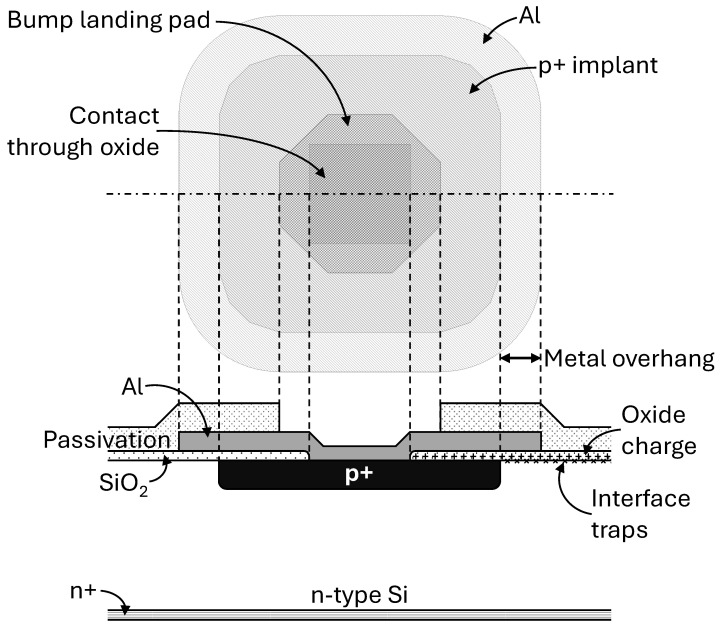
Example of a sensor layout with metal overhang and its associated cross-section. The location of the accumulation of oxide charge and interface traps due to irradiation is shown on the right part of the cross-section. The thickness of the substrate is not to scale with the pixel pitch and should be thicker.

The main contributions on radiation damage (introduced in Section 2) and how the layout influences them are shown in Figure 2.

A typical pixel layout is shown with its related schematic cross-section below. The p+ implant represents the area of the pn-junction, and therefore, the size of the diode (square with rounded corners layout). The Aluminium (Al) layer acts as an electrical connection of the pn-junction to the readout electronics with a flip-chip bump bonding process. The connection is shown as an opening in the SiO_2_ layer (“Contact through oxide”), and the bump-bonding area is shown as “Bump landing pad”. In this specific case, the aluminium layer is larger than the implant size, leading to a “metal overhang”, depleting the interface where the interface traps contribute to the surface leakage current.

**Figure 3:**
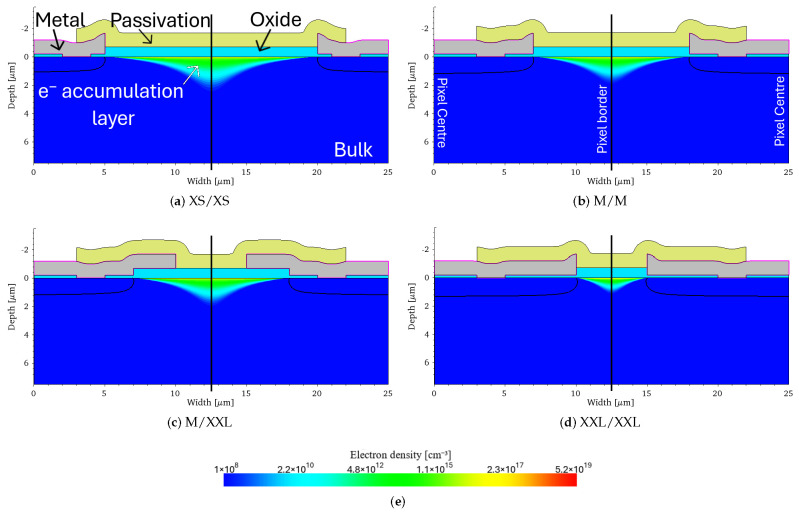
Electron density maps after irradiation to 50 kGy for different layout designs. The maps represent two half pixels centered around the middle of the gap (the limit between two pixels is represented by the black vertical line. The labels of each map correspond to the size of the p+ implant/size of the metal layer as shown in Figure 5. The p+ implants are not visible in the maps, but the TCAD depletion boundary at the pn junction can be observed as the black line below the metal-covered area.

The second microscopic level effect is the generation of oxide charge, together with the charged interface traps, increasing the electron accumulation layer below the interface due to the increased width of the undepleted region [16]. Two-dimensional TCAD simulations have been conducted to observe the electron accumulation effects after irradiation in the inter-implant gap, and the resulting cross sections are shown in Figure 3.

The label of each configuration represents the simulated dimensions of the p-implant and of the metal layer. The size difference between two adjacent variants is 2 µm. The dimensions match the ones of the experimentally tested layouts presented in Section 3. The simulations were conducted for a 320 µm thick n-type silicon sensor (Phosphorus doping concentration: 5 × 10^11^ cm^−3^) biased at 320 V. The radiation damage model for recombination is based on surface Shockley–Read–Hall (SRH) mechanisms, with traps represented by a fixed charge at the Si–SiO_2_ interface. At 50 kGy, the surface recombination velocity was set to 1944 cm·s^−1^, and the oxide charge to 1.9 × 10^12^ cm^−2^ [17].

**Figure 4:**
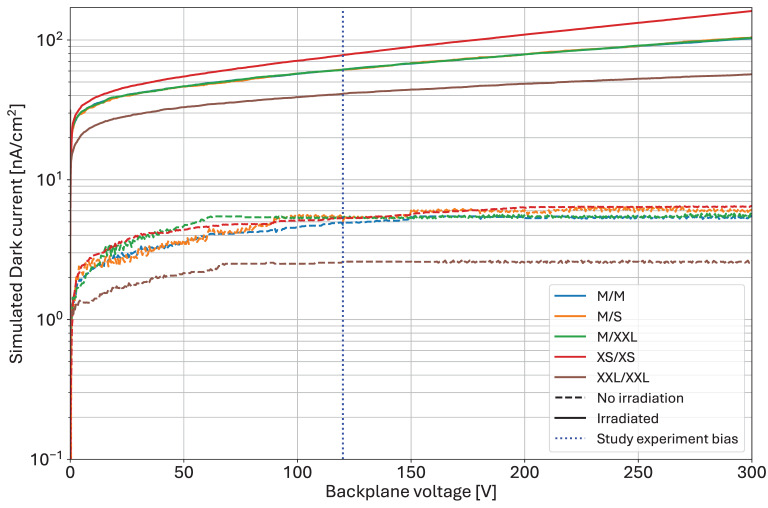
TCAD simulated I-V curves for different inter-implant gaps before (dashed lines) and after a dose of 50 kGy (solid lines). At 0 Gy, the surface recombination velocity was set to 12 cm·s^−1^, and the oxide charge to 2.8 × 10^10^ cm^−2^. At 50 kGy, the surface recombination velocity was set to 1944 cm·s^−1^, and the oxide charge to 1.9 × 10^12^ cm^−2^ [17]. The meaning of the size labels (M/M, M/S, etc.) in the legend will be explained in the next section in detail. They refer to different sizes of implants and metal layers.

The main change with the variation in the implant distance is the size and maximum depth of the electron accumulation layer. In Figure 3a, with the largest gap, the electrons accumulate in a layer with a depth of up to 2.5 µm, while the depth of the layer is limited to about 1.25 µm with the narrowest gap (Figure 3d). The intermediate gap dimensions represented in Figure 3b,c display intermediate results which only differ in the presence of metal overhang. This layout feature does not have any effect on the electron accumulation. From these simulations, the largest accumulation in the large gaps will lead to a higher dark current than with narrower gaps, as shown in simulated I-V curves for the configurations presented in Figure 4.

The simulated variation in the dark current before and after irradiation was conducted for biases ranging between 0 V and 300 V. The experiment presented in the subsequent sections has been conducted at 120 V, represented by the blue dotted line of Figure 4. Before irradiation (dashed lines), the narrow gap offers a smaller dark current. After irradiation to 50 kGy (solid lines), the dark current resulting from the electron accumulation in the different sizes follows the trends observed in Figure 4. The medium implant (M/S, M/XXL) shows average dark current, and the overhang has no influence. A larger implant (XXL/XXL) approximately halves the dark current, while a large gap (XS/XS) makes it worse. Therefore, a layout strategy, from the TCAD perspective, that mitigates the dark current consists of an adequate sizing of the inter-implant distance.

**Figure 5:**
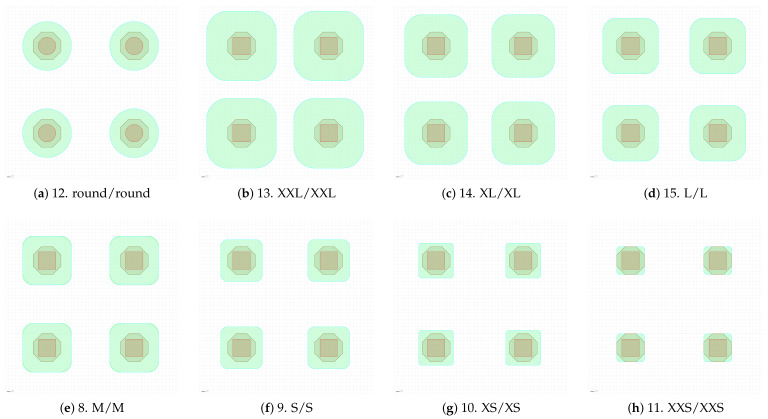

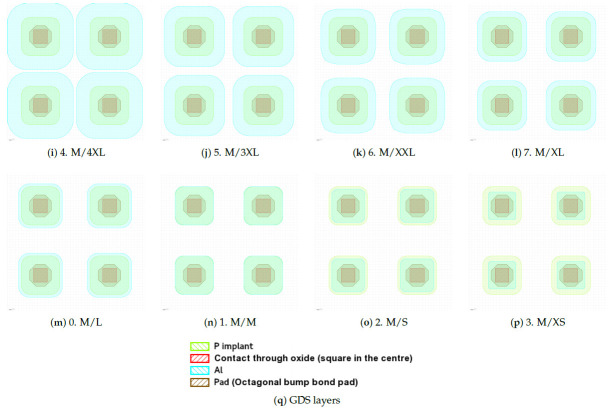
Detailed view of the layout of four pixels for each pixel variant implemented in the prototype sensor. The labels of each variant are composed of the variant number followed by the size of the diode’s p implant, and the size of the metal (p+/Al). The size differs by 2 µm between two adjacent variants (e.g., between S and M).

## 3. Sensor Layout Optimization

To explore the influence of the size of the diode and the metal overhang, a prototype sensor has been designed at the Paul Scherrer Institut and produced by Fondazione Bruno Kessler (FBK, Trento, Italy) [18]. The area of the 320 µm thick sensor is 4 × 4 mm^2^ and contains 16 different pixel flavors with 25 µm pitch arranged in 4 × 4 sub-arrays of 40 × 40 pixels each.

The different designs explore different combinations of diode size and metal overhang configurations. A detailed view of each design is presented in Figure 5. Each variant is numbered from 0 to 15, and the labels represent the size of the p-implant and metal layer sizes, respectively, (p+/Al). The size difference between two adjacent variants (e.g., S and M) is 2 µm.

The average pixel size for this study is represented by the two identical variants 1 and 8 (Figure 5e,n). This design features no overhang: the size of both the diode and the metal is the same (labeled as M).

Variants 9–15 (Figure 5d–f) explore the change in both dimensions to observe the change in behavior when the ratio between the two dimensions is kept constant (without any overhang to isolate the influence of the inter-implant gap). The diode size ranges from XXS (variant 11, Figure 5h) to XXL (variant 13, Figure 5b). It is worth noting that variant 12 (Figure 5a) is different from any other variant as both the implant and the metal are round to further minimize the interpixel capacitance (its diameter corresponds to the M dimension). The round layout and variant 11 (XXS) are expected to offer the lowest capacitance.

The variation in the metal overhang is explored with the remaining variants. Variants 2 and 3 (Figure 5o,p) have a “negative” overhang (metal smaller than the diode) down to size XS. Variants 0, 4–7 (Figure 5i–m) feature an overhang up to size 4XL. All these variants have a diode sized as the default one (M).

Out of these pixel variants, six have been selected as representatives of their design particularities. Four designs with matching dimensions between the diode and the metal were chosen to mainly observe the effects of the diode size, including variant 1 (M, Figure 5n) as an average point; variants 10 and 12 (XS and round, Figure 5a,g), representing small capacitance variants and variant 13 (XXL Figure 5b) with the narrowest inter implant gap. The chosen design with the metal smaller than the diode is variant 2 (M/S, Figure 5o), and the one with metal overhang is variant 6 (M/XXL, Figure 5k). The latter is also the default pixel design outside of this study (e.g., the design of the sensor shown in Figure 1).

Eta distribution histograms [10] have been generated for each of the six pixel designs to evaluate their influence on charge sharing using Cu fluorescence flat fields prior to the irradiation process. All distributions appeared similar, and we can, therefore, conclude that the electric field in small pixel pitches is well set and is not noticeably influenced by the design.

**Figure 6:**
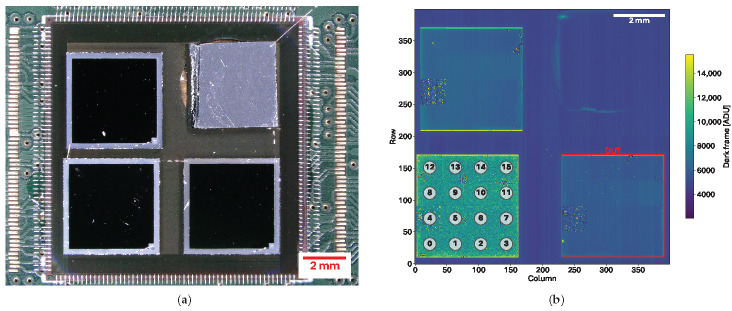
(**a**) Microscope photograph of the three prototype sensors bump-bonded to a MÖNCH 0.3 ASIC. Each sensor has a different backside processing. In the top-right corner, an insensitive piece of Silicon with Aluminum has been glued to allow wire-bonding of the high-voltage connection for sensor biasing. (**b**) Dark frame with 1 ms exposure time of the assembled detector showing the location of the sensor and of the insensitive piece of silicon. Different dark levels appear on each sensor due to the different backside processing. Variant 4 appears defective in all sensors, caused by metal shorting due to the size. The bottom right sensor (with a backside Phosphorus n+ implant) has been chosen for analysis in this study, and is delimited by the red frame.

A set of three of these prototype sensors has been bump-bonded to a single MÖNCH 0.3 ASIC. A photograph of the assembly and its associated dark image is shown in Figure 6.

Each sensor used for this study features a different thin entrance window at the backside processed by FBK (standard sensors do not require such processing). On the photograph (Figure 6a), the difference is noticeable from the slight color change of the metalization ring. The implant of the top left sensor is standard thickness Arsenic n+ doping, the bottom left is ultrashallow Arsenic n+ doping, and the bottom right is standard Phosphorus n+ doping. An insensitive piece of silicon with Aluminum is glued to the top right region to distribute the high-voltage bias to the sensors.

The mean dark frame image (Figure 6b) with an exposure time of 1 ms shows the effect of each implant. Both Arsenic implants exhibit a larger dark current compared to the Phosphorus, with the ultrashallow variant being the worst. Separate studies on these implants have shown similar results. Sensors with the phosphorus implant are already being used for detector systems provided by the PSI CPS detectors group. Since we focus on the anode side optimizations, the results presented hereafter will be taken from the Phosphorus-implanted sensor only.

Another important feature visible in the dark level image is the behavior of variant 4. In all three sensors, the data appear random and do not match the surrounding levels. This is very likely due to the metal deposition being too large, resulting in pixel shorting. The remaining visible defects are mainly associated with bump-bonding-related issues.

## 4. Radiation Damage Evaluation

The chip assembly was wirebonded to its module PCB prior to being installed in a test setup in preparation of the irradiation. Pre-irradiation characterization was conducted to extract the baseline performance. The initial results will be discussed in Section 4.3.

### 4.1. Irradiation Setup

**Figure 7:**
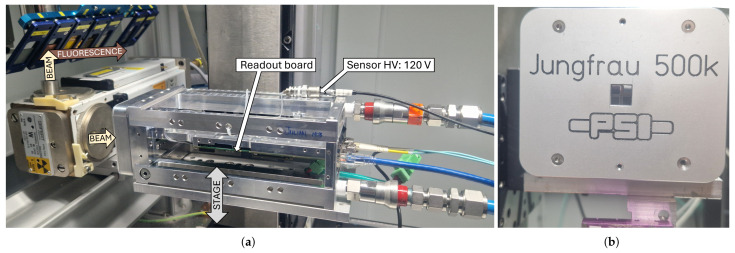
(**a**) Photograph of the test setup in the X-ray test chamber. The chip assembly has been glued and wire-bonded to its carrier PCB and has been installed on the water-cooled mechanics connected to its readout board. The system is on a motorized vertical translation stage to move the sensor either into the direct beam for irradiation or in front of fluorescence targets for calibration. Another position permits placing a reference calibration diode in the direct beam for dose rate calibration. (**b**) Photograph of the detector side of the test system. A thick aluminum cover is used to shield the PCB and passive components. An opening has been made to directly expose the sensor area to the beam. The top part of the reference diode is visible at the bottom.

The detector assembly, comprised of the readout electronics and mechanics, was placed in an X-ray chamber for test and irradiation as shown in Figure 7a.

The entire system was water-cooled mainly to dissipate the heat generated by the readout board electronics and to ensure stable detector performance. The coolant temperature was set to 18 °C, corresponding to a detector temperature of around 27 °C (from separate measurements on an equivalent assembly). The control was ensured with a 100 Mbps RJ45 connection, and the data were streamed out via UDP on a 10 Gbps fibre connection.

The setup was placed in the direct beam of an X-ray tube with a tungsten anode (distance ≈ 12 cm from the anode of the tube) on a vertical motorized translation stage to precisely place either the detector in direct beam (for irradiation) or in front of fluorescence targets (for calibration, the fluorescence element can be chosen and changed using another motorized stage). During operation, the sensor was biased to 120 V. A thick aluminum cover with an opening corresponding to the sensor area was added to shield as much as possible the ASIC’s periphery and the passive components on the carrier PCB, and is visible in Figure 7b.

Another available position of the translation stage permits positioning a 10 µm thick reference calibration diode (Hamamatsu S9724-010, biased at −0.5 V, its top part is visible in Figure 7b) in direct beam for dose rate measurements. The reference diode was aligned with the detector plane to ensure accuracy. At the 12 cm source-detector distance, with the tube settings at 40 kV and 40 mA, the measured dose rate of the direct beam (in the absence of any filter to harden the beam) at the sensor entrance was 100 kGy/h. A simulation of the beam spectrum using SpekPy [19] has been conducted to validate the order of magnitude of the dose rate measurement, and to estimate the dose fraction at several positions in the detector assembly (at the entrance of the sensor, at the diode side of the sensor, and in the ASIC after the bump-bonds). At the sensor entrance, the estimated dose rate is 124 kGy/h, which is within the same order of magnitude of the measurement, with a mean energy of 13.3 keV. On the diode side, the remaining dose rate is 7.5 kGy/h with an increased mean energy of 22.16 keV due to the absorption of low-energy photons in the sensor. Only a small fraction of the initial flux arrived to the ASIC.

### 4.2. Detector Settings, Irradiation and Tests Timings

**Figure 8:**
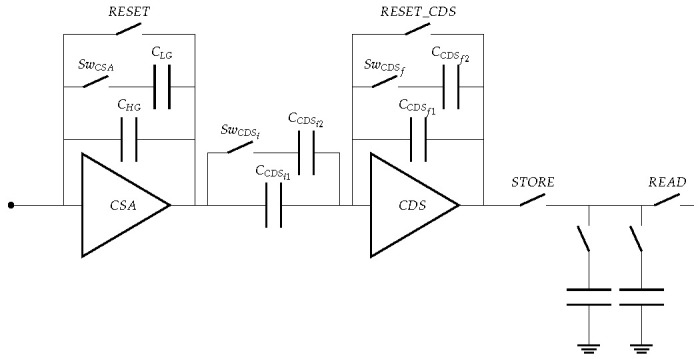
Block schematic of a MÖNCH pixel showing the main capacitors and switches for gain selection and operation. Two different gains can be applied in the charge sensitive amplifier using $Sw_{CSA}$, and three different gains (using 4 configurations with combinations of $Sw_{CDS_{i}}$ and $Sw_{CDS_{f}}$) in the correlated double sampling stage.

The total gain of MÖNCH is adjusted by globally selecting a combination of capacitors in the pixel using switches. A block schematic of the pixel architecture is shown in Figure 8.

The first stage is a Charge Sensitive Amplifier (CSA) where the gain is inversely proportional to the total capacitance of the feedback loop ($C_{HG}+Sw_{CSA}\times C_{LG}$, with $C_{LG}>>C_{HG}$). It is followed by a Correlated Double Sampling (CDS) stage where the gain is given by the ratio of the total capacitance of the input over the total feedback capacitance ($\left(C_{CDS_{i1}}+Sw_{CDS_{i}}\times C_{CDS_{i2}}\right)/\left(C_{CDS_{f1}}+Sw_{CDS_{f}}\times C_{CDS_{f2}}\right)$, with $C_{CDS_{i1,i2}}=2\cdot C_{CDS_{f1,f2}}$). The amplified signal is then stored in either or both storage cells before readout. The gain settings and their capacitors combination used in this work are tabulated in Table 1.

For each step of irradiation, the same testing procedure was initially applied. As soon as the target dose was reached, sets of 1000 dark frames (beam shutter closed) were acquired with increasing integration times (from 1 µs to up to 2 ms) every three minutes for one hour, then every 5 min for another two hours. Immediately after the 3 h of data acquisition ended, the sensor was irradiated to the next dose. The doses (using the diode measurements) at the sensor entrance window were as follows: 0 Gy (pre-irradiation reference), 100 Gy, 500 Gy, 1 kGy, 5 kGy, 10 kGy, 50 kGy, and 100 kGy. According to the SpekPy simulations, the doses at the diode side are about 6% of the sensor entrance window ones. Intermediate data analysis was performed at each step to observe both dark current and noise variations to ensure data quality, and to determine whether the gain settings and/or exposure times needed readjusting. The times and gains used at each dose have been listed in Table 2.

Up to 10 kGy, the pixel gain was set to High Gain/CDS Gain 4 and respected the initial plan of irradiation, followed by three hours of data acquisition. However, the data analysis revealed early signs of saturation at such a high gain due to radiation damage. Therefore, another dataset was acquired around three hours later, alternating between both CDS Gain 1 and 4, with the CSA in high gain. The irradiation to 50 kGy was pursued only 15 h later. After a first dataset acquired at High Gain and CDS Gain 1 and 4, another set was acquired three hours later for both CDS gains at low CSA Gain. Finally, the irradiation to 100 kGy was performed about 88 h later, and a dataset was acquired alternating between High Gain/CDS Gain 4, and Low Gain/CDS Gain 1. Two single datasets have been acquired at several exposure times, and at all gains after 311 h and 506 h, respectively, to observe room temperature annealing dynamics after 100 kGy.

### 4.3. Dark Current and Noise Extraction

**Figure 9:**
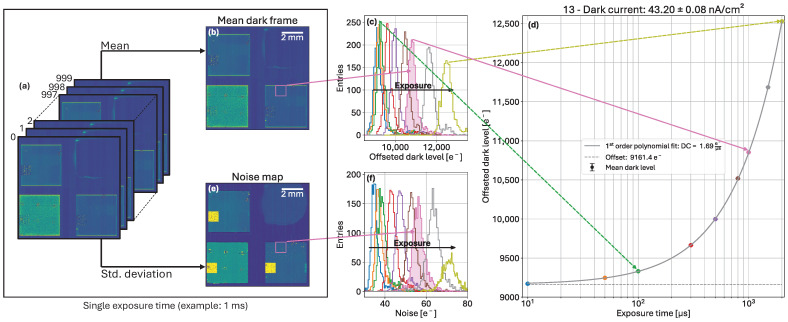
Extraction method of both figures of merit. At different exposure times, a stack of 1000 frames (**a**) is used to compute the mean dark frame (**b**) and the mean noise map (**e**) both shown for 1 ms exposure time. The dark level (**c**) and noise (**f**) histograms are then built and fitted for each pixel design. The mean dark level is then plotted against the exposure time (**d**) to be fitted with a first-order polynomial function to obtain the dark current.

The investigations on the effects of the radiation damage in the sensor bump-bonded to MÖNCH 0.3 were performed using two figures of merit: dark current and noise. The extraction method for both values using the acquired dataset is presented in Figure 9.

For each set of 1000 frames at fixed exposure time (Figure 9a), both the mean dark frame (Figure 9b), and the standard deviation frame (noise map, Figure 9c) are computed. In each map, a histogram for each pixel variant (40 × 40 pixels) in the sensor under test is produced (Figure 9c,f). Gaussian fits are then performed to extract the mean dark level and the mean noise (the value in electrons is converted using the X-ray calibration with monochromatic fluorescence photons). For this, no offset correction has been performed, since it will be taken care of by the fitting procedure later on.

The mean dark level is plotted as a function of exposure time (Figure 9d). A first-order polynomial fit provides the dark level variation per unit time, and can be converted into dark current per unit area using Equation (1):

(1) $$I_{dark}=\frac{a_{fit}\times 1000}{G\times 3.62}\times \frac{1.602\cdot 10^{-19}\times 10^{6}\times 10^{9}}{{\left(25\cdot 10^{-4}\right)}^{2}}\left[nA/cm^{2}\right]$$

with $a_{fit}$ being the mean dark level variation per unit time in ADU/µs obtained from the first order polynomial fit, *G* the conversion gain in ADU/keV obtained from the calibration with fluorescence photons, 3.62 the ionization energy for X-rays in silicon (i.e., the energy needed to create one electron-hole pair) in eV, $1.602\cdot 10^{-19}$ the elementary charge in Coulombs, and ${\left(25\cdot 10^{-4}\right)}^{2}$ the pixel area in cm^2^. All remaining exponent values are for the conversion to nA/cm^2^.

## 5. Results

The noise results are all given for the sensor gain set to the High Gain/CDS Gain 4 with a 10 µs exposure time unless stated otherwise. For the dark current, the gain has been selected to guarantee a sufficient range available for the first-order polynomial fit before saturation, especially at higher doses.

### 5.1. Pre-Irradiation Behavior

**Figure 10:**
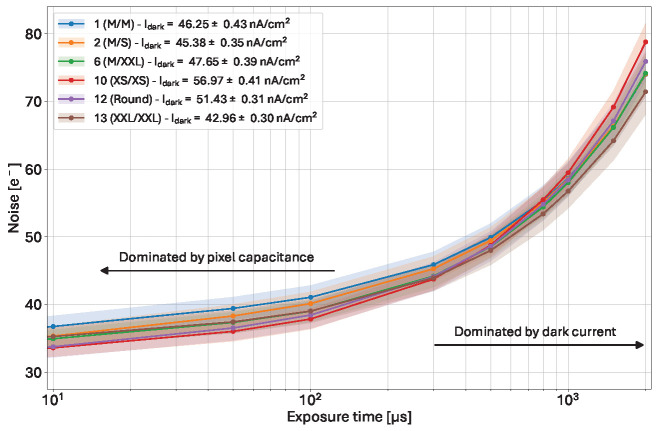
Pre-irradiation noise results of the 6 selected pixel variants against exposure time. The results indicate that the noise is dominated by the pixel capacitance at short exposure times, and by dark current at long exposure times (see text).

Before any irradiation, a reference measurement was performed. The results for the pre-irradiation behavior and the link between noise and dark current are presented in Figure 10.

At short exposure times, the lowest noise is achieved in the variants with the smallest capacitance (10, 12), while the other variants exhibit larger noise. At long exposure times (>500 µs), however, the lowest noise is achieved in the variant with the narrowest inter-pixel gap (variant 13), which is also the variant exhibiting the lowest dark current (42.96 ± 0.30 nA/cm^2^). At the opposite side, variant 10 (large gap) shows both the highest dark current (56.97 ± 0.41 nA/cm^2^) and the highest noise. These results show that the noise is dominated by the pixel capacitance at short exposure times, and by dark current at long exposure times.

### 5.2. Effect of Time After Irradiation

**Figure 11:**
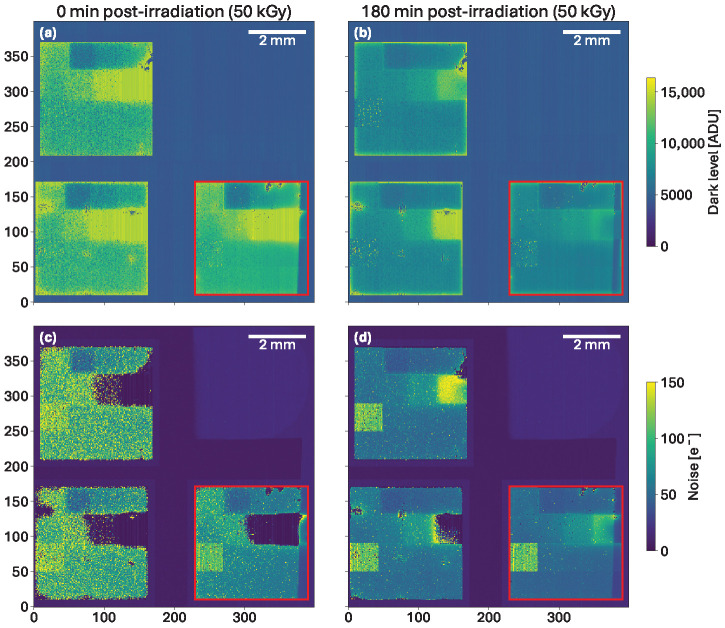
Dark level maps after an accumulated dose of 50 kGy with a 10 µs exposure time directly after the irradiation (**a**), and after 180 min (**b**). Noise maps after an accumulated dose of 50 kGy with a 10 µs exposure time directly after the irradiation (**c**), and after 180 min (**d**). Directly after irradiation, the dark level is close to saturation and the noise levels are very high, while both dark levels and noise values are lower and more uniform after three hours. The tested sensor of this study is the bottom right one, which is delimited by the red frame.

After irradiation, fast short-term annealing occurs and will be explained further in Section 5.5. Such effects present some importance for the dataset analysis. As an example, the dark level and noise maps with an exposure time of 10 µs obtained right after irradiation to 50 kGy, and after 3 h, are shown in Figure 11.

On the left set of images, directly after irradiation, the dark levels (Figure 11a) are either very close to the saturation limit or reaching saturation. The noise levels (Figure 11c) are very high and confirm saturation effects (seen from the almost 0 noise value), especially in variant 11 (XXS/XXS). However, after three hours, the dark level (Figure 11b) is decreased, leading to reduced and more uniform noise levels (Figure 11d). In order to avoid saturation effects, the noise results in the following study are systematically computed from datasets acquired 180 min post-irradiation.

Other visible features can be seen on the noise maps. The first one is that the noise appears to be reduced in the pixels where the ASIC was directly irradiated (due to the absence of sensor coverage; the causes have not been studied). Also, a slanted shadow can be seen on both the dark level and noise maps, on the right side of the detector, due to the cover added to shield the periphery and the passive components on board. Since this shadow also exists within the regions of interest of the sensor chosen for investigation, these shadowed pixels have been excluded from the data analysis.

### 5.3. Effects of Irradiation on the Dark Level

**Figure 12:**
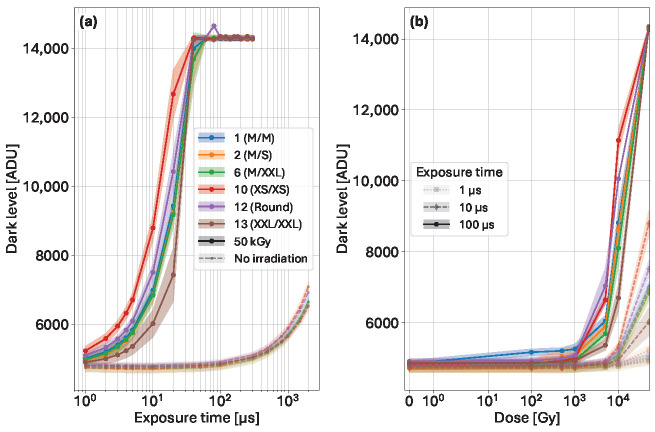
(**a**) Dark level against exposure time before irradiation (dashed lines) and after an accumulated dose of 50 kGy (solid lines). (**b**) Dark level against increasing dose for three exposure times. The signal at 100 µs integration time are saturated.

The effect of the irradiation can be directly seen on the dark level plots. The comparison at different irradiation levels and running conditions with increasing dose on the dark level plots is presented in Figure 12.

Figure 12a shows a direct comparison of the dark level before irradiation and after an accumulated dose of 50 kGy for exposure times ranging from 1 µs to 2 ms with the pixel gains configured in High Gain/CDS Gain 4. Under ideal running conditions, without radiation damage, the dark level slowly increases. At longer exposure times, the dynamic range available remains sufficient to detect single photons in the energy range of interest for such sensors.

After irradiation, however, the dark level increases rapidly with exposure times, reaching saturation for exposure times between 40 µs and 80 µs. Using short exposure times still allows for single-photon detection. When looking directly at pixel variants after irradiation, the design with the narrow implant gap (XXL, variant 13) exhibits better dark level performance than the variant with the small diode (10, XS). This effect is related to the width of the gap; the larger it is, the larger the depleted interface area close to the pixel implant border. With larger gaps, the interface is more difficult to deplete due to the presence of oxide charge.

Figure 12b displays the evolution of the dark level with increasing dose at three different exposure times. At very short exposure times (i.e., 1 µs), the dark level increases very little, leaving ample dynamic range for single photon detection, up to an accumulated dose of 50 kGy. With short exposure times of 10 µs, the effects of irradiation on dark level are more visible above an accumulated dose of 10 kGy, with large variations between designs depending on the pixel gap. Finally, at 100 µs exposure time, all variants display the effects of the damage above an accumulated dose of 1 kGy, being fully saturated at 50 kGy. As previously observed, the larger diode (narrow gap) provides better performance at an equivalent dose compared to the small diodes. From Figure 12 right, it can be seen that the integrated change due to radiation-induced leakage current in the XS/XS design is about 3 times higher than in the variant XXL/XXL.

### 5.4. Dose Effect on Dark Current and Noise

**Figure 13:**
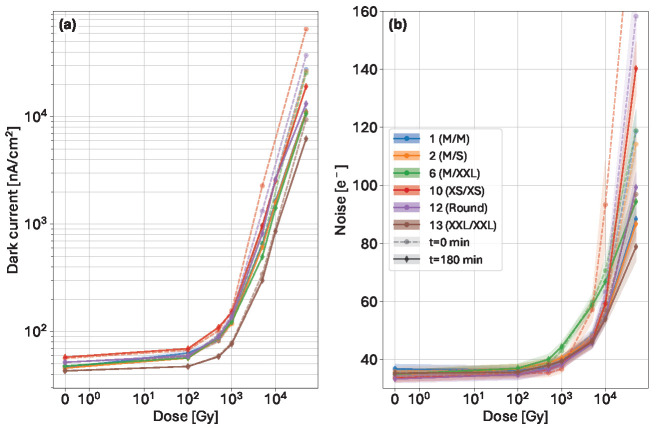
Dark current (**a**) and noise (**b**) (10 µs exposure time) against increasing accumulated dose right after irradiation (dashed lines), and after 180 min (solid lines).

The evolution of both dark current and noise against accumulated dose immediately after irradiation and after 180 min is shown in Figure 13.

The dark current at the highest irradiation dose shows a difference of at least a factor of 5 between XXL/XXL and XS/XS, and a difference of a factor of 2 is observed in the noise values. The dark current (Figure 13a) increases much faster with the dose in designs using larger inter-pixel gaps compared to the values before irradiation. The leakage current has increased by at least 2–3 orders of magnitude. These designs, however, show a quick change in the dark current within the three-hour period after the irradiation. The current variants 10 (XS/XS), and 12 (round) are reduced by 71% and 65%, respectively. All medium-sized diode variants have a reduction of around 59%, and of 34% in the narrow gap variant (13, XXL). The recovery remains insufficient to restore to pre-irradiation values.

The noise plot (Figure 13b) shows similar behavior of increasing values with dose, and recovery after the three-hour time period, but only at the highest dose of 50 kGy. The variant with metal overhang shows a steeper increase in noise with respect to the variants with the same diode size with different overhang configurations hinting adverse effect of the overhang. On the other hand, no clear evidence shows the benefit of having the metal smaller than the implant compared to both having the same size.

Right after irradiation at 50 kGy, the highest noise was observed in the variant with the smallest diode (10, XS/XS), but decreased by around 50% within three hours. For doses below 10 kGy, the round diode of variant 12 displays the lowest noise overall, hinting at shape-related effects, and features the second largest reduction of noise over the time period by around 37.5%. The noise of the narrowest gap design is reduced by around 19% within the same time period.

### 5.5. Annealing

Another important aspect guiding the design of the pixel is the evolution of both figures of merit after the irradiation has been carried out. The room-temperature annealing effects can be observed over two time scales: short (O (minutes)), and long (O (days to years)). Effects of annealing at higher temperatures were not explored in this work.

#### 5.5.1. Short Term Annealing

**Figure 14:**
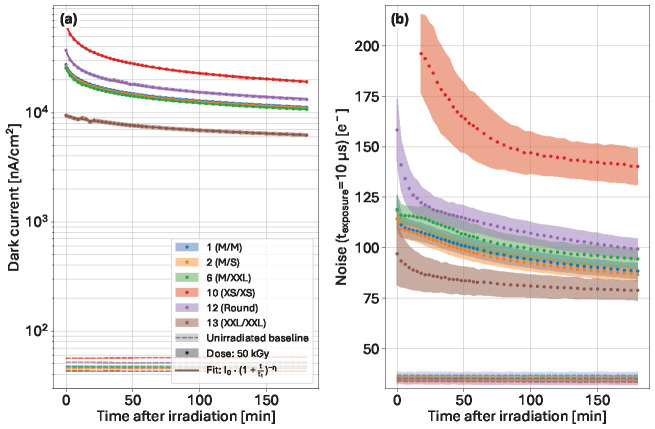
Evolution of dark current (**a**) and noise (**b**) (10 µs exposure time) during the first 180 min following irradiation to a dose of 50 kGy.

In the previous sections, short-term annealing effects have been considered for the data analysis after the observation of the noise levels right after the end of the irradiation, and after 180 min. The dynamics can be observed within the acquired dataset, composed of both dark current and noise data obtained at regular intervals. The short-term effects over the 180 min period after irradiation to an accumulated dose of 50 kGy are presented in Figure 14.

The dark current (Figure 14a) follows a similar decreasing trend for all the variants towards a stable value. The increase in the dark current with respect to the unirradiated measurement is between 2 and 3 orders of magnitude higher. As previously seen, the small diode variant exhibits a higher dark current than the variant with the narrow implant. The evolution of the dark current with time after irradiation for each studied design can be expressed with Equation (2) [20]:

(2) Isurf(t)=I0·(1+tt1)−η

with $I_{0}$ being the dark current as soon as the irradiation process ends (simplified to be at t = 0, neglecting the real time between the shutter closing and the actual start of the measurement), $t_{1}$ the time constant (temperature dependent [20]), and η an exponential factor related to the ratio of reaction rates.

The dark current as a function of annealing time has been fitted using Equation (2). The obtained mean value of η ($\bar{\eta }=0.22\pm 0.04$) matches well with the obtained parameter in [20]. However, the average value of $t_{1}$ ($\bar{t_{1}}=3.38\pm 0.44$ min, with variant 13 excluded) is very different, which is due to the large change in temperature between both experiments. The measurements in [20] have been conducted at 60 °C and 80 °C, while the present study is at 27 °C. At a very short exposure time (10 µs), the noise levels (Figure 14b in all variants decrease with the dark current.

The average size design (1, M/M) and its variant with negative metal overhang (2, M/S) have very similar trends and noise values (from 115 e^−^ directly after irradiation to 88 e^−^ three hours later). The variant with metal overhang (6, M/XXL) displays noticeably higher noise with an equivalent decreasing rate trend (from 119 e^−^ to 94 e^−^). Both variants featuring the lowest capacitance designs exhibit the highest noise. The round design (12) noise after irradiation is 160 e^−^, which decreases at a faster rate for the first half hour than for the M-sized diodes, then steadily decreases to reach 99 e^−^ after three hours. A similar trend is visible in the XS/XS variant (10), where the initial noise could not be computed due to saturation. The noise value of the smallest feature does not decrease as much as the other variants and remains limited to 140 e^−^ after the short annealing period. Finally, the small gap variant (13; XXL/XXL) also rapidly decreases over the first half hour to then display a much slower reduction from 97 e^−^ down to 79 e^−^. Nonetheless, neither the dark current nor the noise decrease with sufficiently high rates to reach unirradiated baseline values.

**Figure 15:**
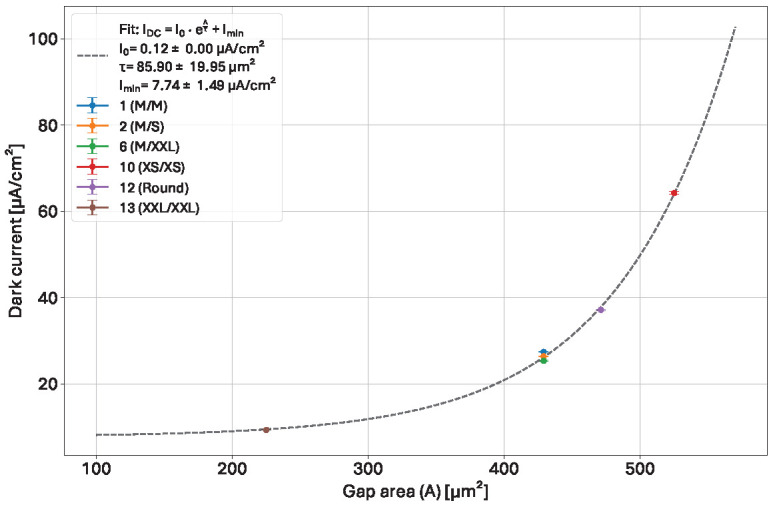
Dark current directly after irradiation obtained from fitting the data of Figure 14 with Equation (2) against the gap area.

The dark current values directly after irradiation extracted from the data in Figure 14 left from the fitting with Equation (2) have been plotted against the gap area (neglecting the rounded corners of the square diodes) in Figure 15 and fitted using Equation (3):

(3) IDC=I0·eAτ+Imin

with *A* the gap area, τ the area scaling factor, $I_{0}$ the pre-exponential factor, and $I_{min}$ the baseline dark current.

The dark current increases exponentially with the area of the gap. With a sufficiently small gap area, the minimum achievable dark current after irradiation in this example at 50 kGy would be 7.74 ± 1.49 µA/cm^2^. The dark current value of the large implant version (13; XXL/XXL) is very close to this lower limit. The benefits of reducing the gap further would nonetheless be negligible when technologically possible (risks of shorting in larger features as seen with the 4XL metal variant).

#### 5.5.2. Long Term Annealing

**Figure 16:**
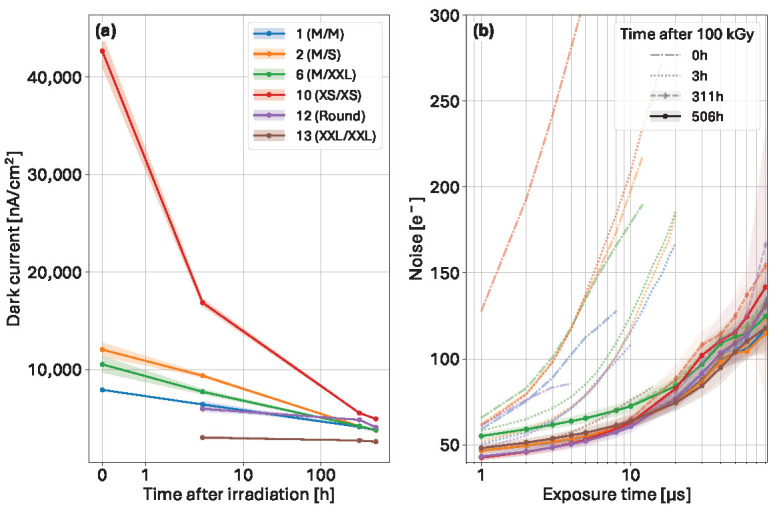
(**a**) Evolution of the dark current over 506 h following irradiation to a dose of 100 kGy. (**b**) Noise against exposure time directly after irradiation (dash-dotted lines), 3 h after (dotted lines) displaying early signs of saturation, 311 h after (dashed lines with diamond markers), and 506 h after (solid lines with circle markers) irradiation to a dose of 100 kGy.

The effects of longer annealing time scales, up to over 500 h after irradiation at 100 kGy, are presented in Figure 16.

At the highest accumulated dose for the study (100 kGy), the dark current (Figure 16a) directly after the irradiation process could not be extracted for some variants (12; round, 13; XXL/XXL) due to rapid saturation even at shorter exposure times. For these two designs, especially the round one, even the extracted value 3 h post-irradiation appears to be wrong. Nonetheless, the global dynamics follow similar trends seen over shorter time scales, and the effects of the gap area are similar. However, the annealing dynamics of the dark current at longer annealing times show a different behavior, mainly due to the annealing dynamics of both the oxide charge and the interface traps working together, which cannot follow Equation (2) anymore.

The noise plot (Figure 16b) shows the level of improvement that can be achieved after room temperature annealing when comparing the noise directly after irradiation (transparent dash dotted lines), and 3 h later (transparent dotted lines). In both cases, the noise quickly increases with exposure time and then shows signs of pixel saturation. After several days of room-temperature annealing, the rate of increase in noise with exposure time is reduced.

At short exposure times, the variant with metal overhang (6, M/XXL) exhibits the highest noise, and the low interpixel capacitance designs (10, XS/XS; 12, round) show the lowest. At longer exposure times (>20 µs), the noise becomes higher in the latter. On the other hand, at these times, the variants without metal overhang and negative overhang show the lowest noise. The trends also suggest that the latter variant (2, M/S) could offer better noise performance than the equivalent size counterparts.

## 6. Conclusions

With the upgrade of synchrotron light sources to diffraction-limited storage rings, a large increase in brilliance is expected (up to three orders of magnitude at some beamlines of SLS 2.0). The increased photon flux will, in turn, lead to greater risks of radiation damage in sensors of hybrid pixel detectors. Evidence of radiation-induced damage has already been observed, among others, in sensors bump-bonded to MÖNCH 0.3, a 25 µm charge integrating HPD as reported.

Two main contributors to adverse effects can be linked to design features of the sensor pixel. The first contributor is the increase in the oxide charge, leading to an increased electron accumulation layer below the interface, which can be mitigated by optimizing the gap between the two implants. The second is the increase in the interface traps leading to an increase in the surface leakage current at the depleted interface, in particular, below the metal layer overhanging beyond the implant, which can be adjusted by design.

A 4 × 4 mm^2^ sensor prototype composed of 16 different pixel variants with 25 µm pitch was designed at the Paul Scherrer Institut and fabricated by the Fondazione Bruno Kessler to explore the effect of the diode size and of the metal overhang on radiation damage. Three sensors have been bump-bonded to a MÖNCH 0.3 ASIC. The sensor has been tested and irradiated in a laboratory at a dose rate of 100 kGy/h up to 100 kGy at the sensor entrance window side with regular probing to observe the effects on six chosen pixel designs.

The variant with the regular diode size and the smaller metal layer (2, M/S) exhibits low noise at high dose and continues to display the lowest noise level after annealing (of the interface traps) at longer exposure times. In the structure with metal overhang (6, M/XXL), which is the group’s default pixel design for sensors outside of this study, the dark current remains within the same range as the other variants with the same diode size. However, the noise levels of this variant are the highest of all designs after irradiation due to the increased leakage current at the depleted interface below the overhang caused by the augmentation of interface traps. No significant improvement can be observed with annealing. The designs without metal overhang featuring the same diode size display better noise performance. The variants with the largest inter-implant gap, with the lower capacitance (10, XS/XS; 12 round), offer the best noise performance at short exposure times before and after irradiation. However, for these designs, the noise is the highest of all variants at long exposure times and after irradiation due to the large surface leakage current. The noise also rises quickly after irradiation but recovers faster after annealing than for any other variant, with the evacuation of excess electrons accumulated at the interface. The noise performance of the round variant is overall the best. Finally, the variant with both the large implant and the large aluminium coverage offers the lowest dark current due to the limited increase in the oxide charge within the reduced pixel gap area. At long exposure times, this design offers better noise performance as it becomes dominated by dark current. The operation appears very stable with time directly after irradiation. Therefore, to improve radiation hardness by design, the current default pixel variant (M/XXL) should be changed to incorporate a larger diode (XXL) without metal overhang.

The irradiation was conducted at a very high dose rate with respect to the ones experienced at synchrotron beam lines. A further study could be conducted at more realistic rates (O (100 Gy/h)) to observe the changes in the radiation damage dynamics. Lower dose rates could lead to higher radiation damage for similar doses with respect to this study. Another aspect of the irradiation dynamics that merits study is the high temperature annealing effects (e.g., at 60–80 °C). The noise measurements were all conducted at short exposure times to be able to obtain exploitable data at higher doses to avoid quick saturation effects. Such durations do not reflect the typical use at a synchrotron beamline. A full duty cycle with MÖNCH is achieved with 170 µs exposure times.

From the results obtained in this study, further refinements of the best design could be studied in a future iteration, such as optimized large-size diodes (small inter-pixel gap) and different metal coverage (smaller than the implant). The round design could also be tested with several variations in diameter and metal size combinations. However, the new design strategies are, by themselves, insufficient in the pursuit of detectors that are sufficiently radiation hard to withstand the expected increased brilliance at fourth-generation synchrotrons. Improvements can be achieved with X-ray tailored modifications to the sensor process, such as optimized dielectric layer, thicknesses, and surface treatments to minimize charge accumulation. Additionally, if very low noise at long exposure times and high doses is required, a focus must be put on cooling (<−20 °C) the detector to drastically reduce the leakage currents and their contribution to the overall noise.

## Figures and Tables

**Table 1:** Bit states summary for the gain configurations of MÖNCH 0.3 in this work. A CDS gain of 2 is also available from two independent bit configurations, but was not used.

Gain	${\mathrm{Sw}}_{\mathrm{CSA}}$	${\mathrm{Sw}}_{{\mathrm{CDS}}_{i}}$	${\mathrm{Sw}}_{{\mathrm{CDS}}_{f}}$
High Gain/CDS Gain 1	0	0	1
High Gain/CDS Gain 4	0	1	0
Low Gain/CDS Gain 1	1	0	1
Low Gain/CDS Gain 4	1	1	0

**Table 2:** Summary of the time elapsed during the experiment. For each dose, the time of data acquisition and its related gain settings are given. Up to 10 kGy, the irradiation and data acquisition followed each other. After preliminary analysis, gains were then readjusted to extend the dynamic range following radiation damage.

Elapsed Time [h]	Dose [Gy]	Gain Settings
0–3	Unirradiated	High Gain/CDS Gain 4
3–6	100	High Gain/CDS Gain 4
6–9	500	High Gain/CDS Gain 4
9–12	1k	High Gain/CDS Gain 4
12–15	5k	High Gain/CDS Gain 4
15–18	10k	High Gain/CDS Gain 4
21–24		High Gain/CDS Gain 4, High Gain/CDS Gain 1
39–42	50k	High Gain/CDS Gain 4, High Gain/CDS Gain 1
45–48		Low Gain/CDS Gain 4, Low Gain/CDS Gain 1
136–139	100k	High Gain/CDS Gain 4, Low Gain/CDS Gain 1
450		All configurations (single acquisition)
645		All configurations (single acquisition)

## Data Availability

The data is available upon request.

## Abbreviations

The following abbreviations are used in this manuscript:

ADU	Analog-to-Digital Unit
ASIC	Application-Specific Integrated Circuit
CDS	Correlated Double Sampling
CPS	center for Photon Science
CSA	Charge-Sensitive Amplifier
DLSR	Diffraction-Limited Storage ring
ENC	Equivalent Noise Charge
FBK	Fondazione Bruno Kessler
HPD	Hybrid Pixel Detector
PCB	Printed Circuit Board
PSI	Paul Scherrer Institut
SLS	Swiss Light Source
SRH	Shockley-Read-Hall
XFEL	X-ray Free Electron Laser
